# Wnt5a-induced cell migration is associated with the aggressiveness of estrogen receptor-positive breast cancer

**DOI:** 10.18632/oncotarget.24761

**Published:** 2018-04-20

**Authors:** Yoshie Kobayashi, Takayuki Kadoya, Ai Amioka, Hideaki Hanaki, Shinsuke Sasada, Norio Masumoto, Hideki Yamamoto, Koji Arihiro, Akira Kikuchi, Morihito Okada

**Affiliations:** ^1^ Department of Surgical Oncology, Research Center for Radiation Casualty Medicine, Research Institute for Radiation Biology and Medicine, Hiroshima University, Minami-ku, Hiroshima, 734-8551, Japan; ^2^ Department of Molecular Biology and Biochemistry, Graduate School of Medicine, Osaka University, Suita, Osaka, 565-0871, Japan; ^3^ Department of Anatomical Pathology, Hiroshima University Hospital, Minami-ku, Hiroshima, 734-8551, Japan

**Keywords:** Wnt5a, ER-positive breast cancer, cell migration, activated leukocyte cell adhesion molecule, prognosis

## Abstract

Elevated expression of Wnt5a is associated with malignancy, cell invasion, and metastasis. The role of Wnt5a expression in breast cancer remains elusive. We investigated the significance of Wnt5a expression in breast cancer. The relationship between Wnt5a expression and clinicopathologic factors was assessed in invasive breast cancer (*n* = 178) resected at Hiroshima University Hospital between January 2011 and February 2014. Wnt5a was expressed in 69 of 178 cases (39%) of invasive breast cancer and correlated strongly with estrogen receptor (ER) expression (*P* < 0.001). Wnt5a expression in ER-positive breast cancer correlated significantly with lymph node metastasis, nuclear grade, and lymphatic invasion. The recurrence-free survival was shorter in breast cancer patients with Wnt5a expression than in those without (*P* = 0.024). The migratory capacity of ER-positive breast cancer cells increased with constitutive expression of Wnt5a and decreased with Wnt5a knockdown. DNA microarray analysis identified activated leukocyte cell adhesion molecule (ALCAM) as the primary gene induced by Wnt5a. ALCAM was expressed in 69% of Wnt5a-positive but only 27% of Wnt5a-negative cancers (κ = 0.444; *P* < 0.001). The inhibition of ALCAM reversed the enhanced migratory effect of Wnt5a, confirming the importance of this protein in the migration of ER-positive breast cancer cells. Wnt5a expression is related to high malignancy and a poor prognosis in ER-positive breast cancer. We suspect that Wnt5a expression increases the malignancy of breast cancer by increasing the migratory capacity of cancer cells through the induction of ALCAM expression.

## INTRODUCTION

Wnt signaling occurs in β-catenin-dependent pathways, through β-catenin regulates the expression of many genes, and in the β-catenin-independent planar cell polarity and Wnt/Ca^2+^ pathways [1, 2]. β-catenin-dependent pathways are involved in some cancers, for example, familial polyposis, in which the adenomatous polyposis coli mutation causes the accumulation of β-catenin in the nucleus, resulting in colorectal cancer [2, 3]. In β-catenin-independent pathways, Wnt5a is a representative activating ligand involved in cell motility and cell polarity through downstream signaling (e.g., JNK phosphorylation) [4]. Wnt5a expression correlates significantly with malignancy and stage of progression in malignant melanoma [5, 6], gastric cancer [7–9], prostate cancer [10], lung cancer [11], and pancreatic cancer [12]. In contrast, Wnt5a is a tumor suppressor in colorectal cancer [13], thyroid cancer [14], liver cancer [15], and malignant lymphoma [16]. Thus, the effect of Wnt5a expression differs between organs.

Whether Wnt5a expression in breast cancer correlates with pathological factors such as lymph node metastasis and grade is unknown. While a significant correlation between the immunohistochemical disappearance of Wnt5a expression and poor prognosis [17, 18] has been reported, these studies do not distinguish between breast cancers subtypes. Breast cancer is classified into four subtypes based on the expression of the estrogen receptor (ER) and human epidermal growth factor receptor 2 (HER2) [19, 20]. Because ER-positive and -negative breast cancers derive from different tissues (ductal epithelium and basal cells, respectively), the two subtypes are biologically distinct. ER-positive breast cancers progress slowly, whereas those that are ER-negative are highly malignant and have a poor prognosis. In previous studies, the inclusion of all subtypes, with their remarkably distinct prognoses, likely interfered with the accurate determination of the role of Wnt5a in breast cancer.

A positive correlation is reported between Wnt5a expression level and ER-positive breast cancer, as determined using breast cancer specimens [17, 18]. A correlation was observed between the *PIK3CA* mutation and Wnt5a expression based on the examination of 43 cases of ER-positive tumors [21]. Another study reports no significant correlation between the expression of Wnt5a and ER status, as determined by examination of 94 stained breast cancer specimens [22]. We believe that to determine the role of Wnt5a expression in breast cancer, the level of Wnt5a expression in each of the breast cancer subtypes must first be determined.

The mechanism of malignant transformation by Wnt5a has been studied in a variety of cancers. Cell motility in Wnt5a-positive gastric cancer is increased through activation of FAK and Rac to induce malignant transformation [7, 8]. One study of breast cancer reports an increase in expression of Wnt5a/b and their respective receptors Ror1 and 2 in brain metastases [23]. Other studies report that Wnt5a expression increases the malignancy of breast cancer through activation of tumor-related macrophages [24] or the promotion of cell migration [25]. However, to date, no study has described the mechanism underlying malignant transformation by Wnt5a in breast cancer.

In this study, we examine the significance of Wnt5a expression in breast cancer by determining the clinicopathologic characteristics of Wnt5a-positive breast cancers using Wnt5a immunohistochemical analysis of breast cancer specimens. We investigate the mechanism of malignant transformation in breast cancer by Wnt5a through biological analyses of cultured cells.

## RESULTS

### Wnt5a is expressed in ER-positive breast cancer

**Figure 1:**
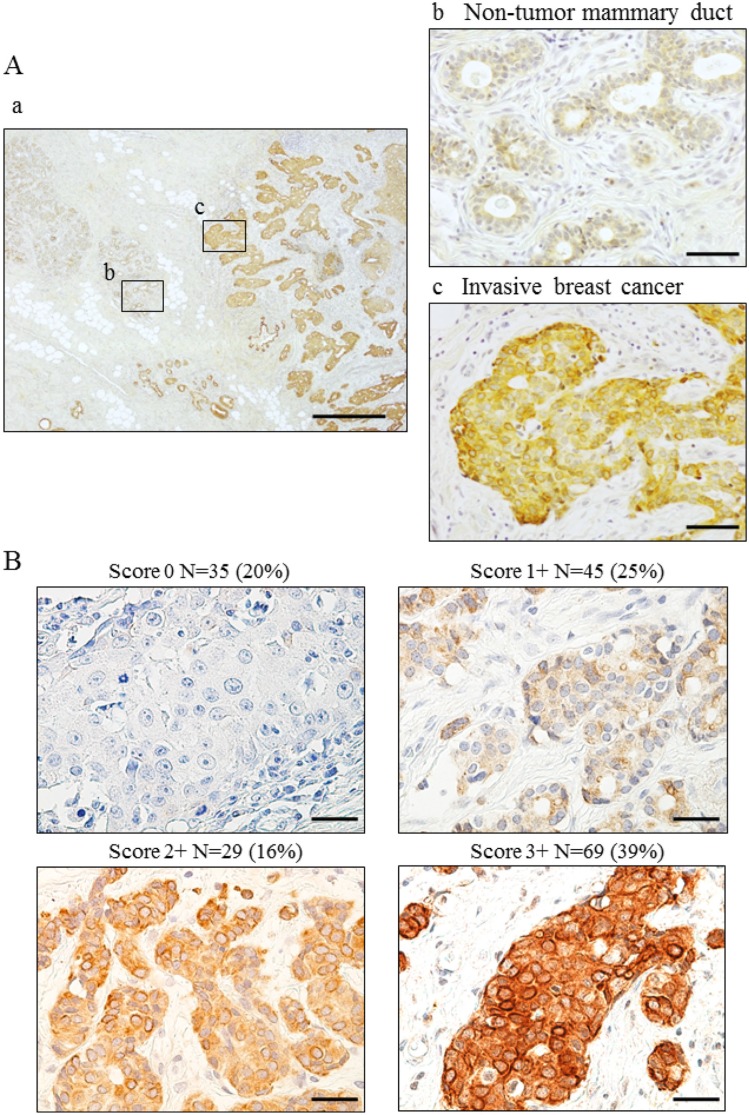
Wnt5a expression in breast cancer using breast cancer specimens (**A**) Immunohistochemical staining for Wnt5a in invasive breast cancer. (a) Wnt5a expression in invasive breast cancer. (b) Wnt5a expressed weakly in cytoplasm in non-tumor mammary duct. (c) Wnt5a expressed strongly in invasive breast cancer a: bar, 500 μm, magnification ×40; b, c: bar 50 μm, ×400 (**B**) Evaluation of Wnt5a expression was scored as 0, 1+, 2+, or 3+, taking into consideration both staining proportion and intensity. Scores 0–2+, negative; Score 3+, positive. bar, 50 μm, ×400.

We observed weak Wnt5a expression in non-tumor ductal epithelial cells but none in basal or stromal cells (Figure 1Aa, 1Ab). Wnt5a was expressed in the cytoplasm but not the nucleus of breast cancer cells (Figure 1Aa, 1Ac). Wnt5a expression scores are shown in Figure 1B. Scores of 0, 1+, 2+, and 3+ accounted for 20%, 25%, 16%, and 39% of the specimen scores, respectively. A score of 3+ was defined to be positive for Wnt5a expression.

Of the 178 cases of invasive breast cancer, 69 (39%) were Wnt5a-positive and 109 (61%) were Wnt5a-negative (Table 1, Supplementary Table 1). A very strong correlation was observed between Wnt5a expression and positivity for ER or PgR. Wnt5a expression was extremely low in ER-negative breast cancers. There was no correlation between Wnt5a expression and HER2 positivity. Wnt5a-positive breast cancers were classified into subtypes based on ER status, as shown in Table 1.

**Table 1:** Relation of Wnt5a expression with ER, PgR and HER-2 in breast cancer

		Wnt5a expression		
	Total (*n* = 178)	Negative (*n* = 109)	Positive (*n* = 69)	*P* value
ER, *N* (%)				
Negative	25 (14)	24 (22)	1 (1)	
Positive	153 (86)	85 (78)	68 (99)	<0.001
PgR, *N* (%)				
Negative	38 (21)	34 (31)	4 (6)	
Positive	140 (79)	75 (69)	65 (94)	<0.001
HER2, *N* (%)				
Negative	159 (89)	96 (88)	63 (91)	
Positive	19 (11)	13 (12)	6 (9)	0.496

### Wnt5a expression is associated with high-grade malignancy in ER-positive breast cancer

**Figure 2:**
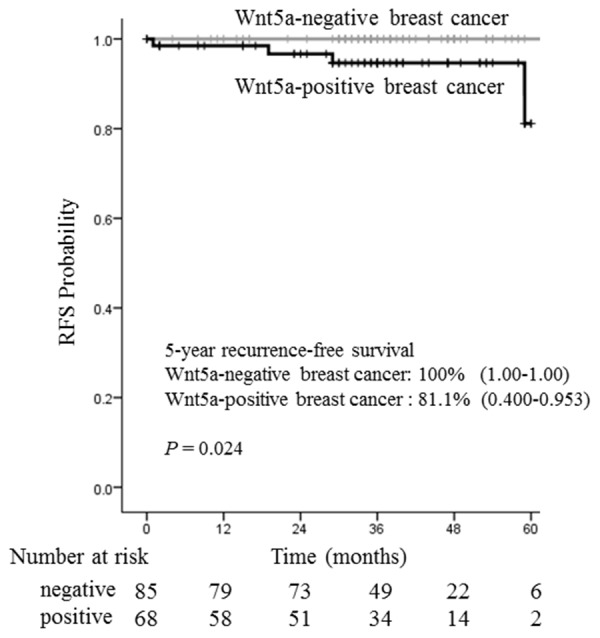
Relationship between recurrence-free survival and Wnt5a in 153 ER-positive breast cancer patients The recurrence-free survival rates were analyzed according to Wnt5a immunohistochemistry. The mean 5-year recurrence-free survival rates were 100% for Wnt5a negative breast cancer and 81.1% for Wnt5a negative breast cancer. Wnt5a expression was significantly associated with poor recurrence-free survival in ER-positive cancer patients (*P* = 0.024).

Because we observed that Wnt5a expression is associated with ER-positive breast cancer, we investigated the pathological factors and prognosis of ER-positive breast cancers exclusively. Analysis of 153 ER-positive cases revealed a significant difference between Wnt5a-positive and negative breast cancer in the presence of lymph node metastasis (*P* < 0.001), nuclear grade (*P* = 0.004), and lymphatic invasion (*P* = 0.002) (Table 2). Although no significant correlation was seen, there were clear trends toward a relationship between Wnt5a expression and the presence of vessel invasion (*P* = 0.050), tumor size (*P* = 0.069), and Ki-67 labeling index (*P* = 0.058). These data suggest that malignancy is higher in Wnt5a-positive breast cancer that is also ER-positive rather than ER-negative. Comparison of recurrence-free survival, for which 5-year survival rates were 81.1% and 100% in Wnt5a-positive and Wnt5a-negative breast cancers, respectively, revealed a significant difference according to statistical analysis using the log-rank test (*P* = 0.024) (Figure 2). The clinicopathological factors and recurrence sites of ER-positive breast cancer are shown in Supplementary Table 2.

**Table 2:** Relation between Wnt5a expression and clinicopathological factors in ER-positive breast cancer

		Wnt5a expression		
	Total (*N* = 153)	Negative (*n* = 85)	Positive (*n* = 68)	*P* value
Age (median, range)		63, 35–86	57.5, 34–87	0.065
Age, *N* (%)				
≤45	28 (18)	13 (15)	15 (22)	
>45	125 (82)	72 (85)	53 (78)	0.282
Menopausal status, *N* (%)				
Premenopausal	58 (38)	27 (32)	31 (46)	
Postmenopausal	95 (62)	58 (68)	37 (54)	0.080
Tumor size, *N* (%)				
pT1 ≤ 20 mm	104 (44)	63 (74)	41 (60)	
pT2/pT3 > 20 mm	49 (56)	22 (26)	27 (40)	0.069
Lymph node metastasis, *N* (%)				
Negative	103 (67)	72 (85)	31 (46)	
Positive	50 (33)	13 (15)	37 (54)	<0.001
Nuclear grade, *N* (%)				
1/2	85 (56)	56 (66)	29 (43)	
3	68 (44)	29 (34)	39 (57)	0.004
Lymphatic invasion, *N* (%)				
Negative	101 (66)	65 (76)	36 (53)	
Positive	52 (34)	20 (24)	32 (47)	0.002
Vessel invasion, *N* (%)				
Negative	142 (93)	82 (96)	60 (88)	
Positive	11 (7)	3 (4)	8 (12)	0.050
Ki-67, *N* (%)				
0–20	67 (79)	43 (51)	24 (35)	
21–100	86 (56)	42 (49)	44 (65)	0.058

### Migratory capacity of Wnt5a-expressing breast cancer cells

**Figure 3:**
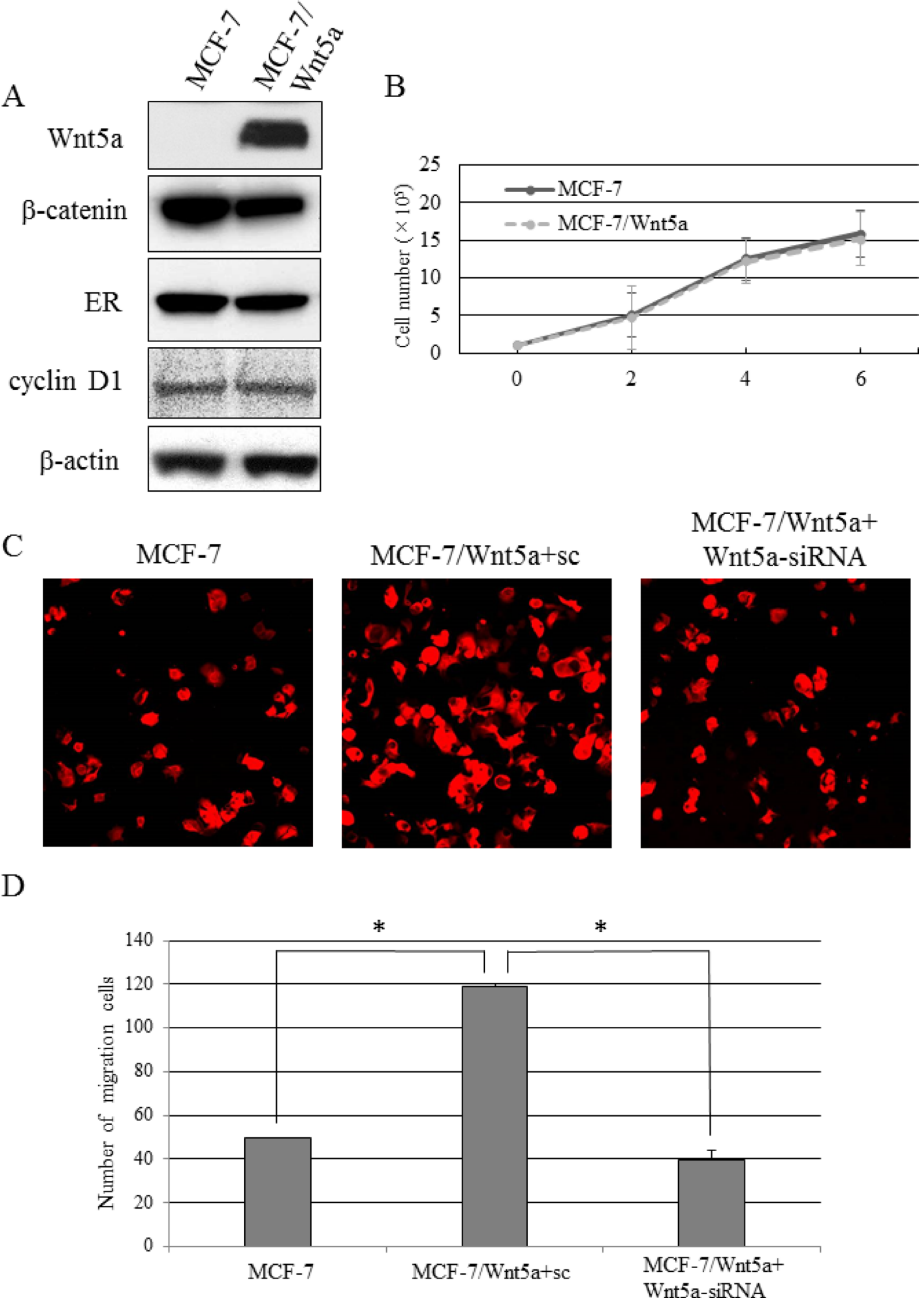
Migratory capacity of Wnt5a-expressing breast cancer cells (**A**) The expression levels of β-catenin, ER, and cyclin D in MCF-7/Wnt5a and control MCF-7 cells. (**B**) The proliferative capacity in MCF-7/Wnt5a and control MCF-7 cells. Data are presented as the mean ± SE of three proliferation assays. (**C**, **D**) The migratory capacity of MCF-7, MCF-7/Wnt5a + sc, and MCF-7/Wnt5a + Wnt5a-siRNA cells. Data are presented as the mean ± SE of three migration assays. Data were evaluated using the Mann–Whitney *U*-test.

We prepared MCF-7, an ER-positive breast cancer cell line expressing no Wnt5a, and forced it to express Wnt5a constitutively (MCF-7/Wnt5a cells). These cells showed no change in the levels of proteins involved in breast cancer–related signaling pathways or β-catenin-dependent pathways, such as ER, β-catenin, and cyclin D1 (Figure 3A). There was no significant difference in the proliferative capacity between MCF-7/Wnt5a and control MCF-7 cells (Figure 3B). As Wnt5a is reported to associate with cell migration, we conducted cell migration assays to determine the migratory capacity of Wnt5a-positive breast cancer cells. We also tested Wnt5a-silenced MCF-7/Wnt5a cells (MCF-7/Wnt5a + Wnt5a-siRNA cells), bringing the total number of cell types investigated to three. The migratory capacity of MCF-7/Wnt5a cells increased significantly 12 h later, whereas knockdown of Wnt5a resulted in a lower increase in migratory capacity (Figure 3C, 3D).

**Figure 4:**
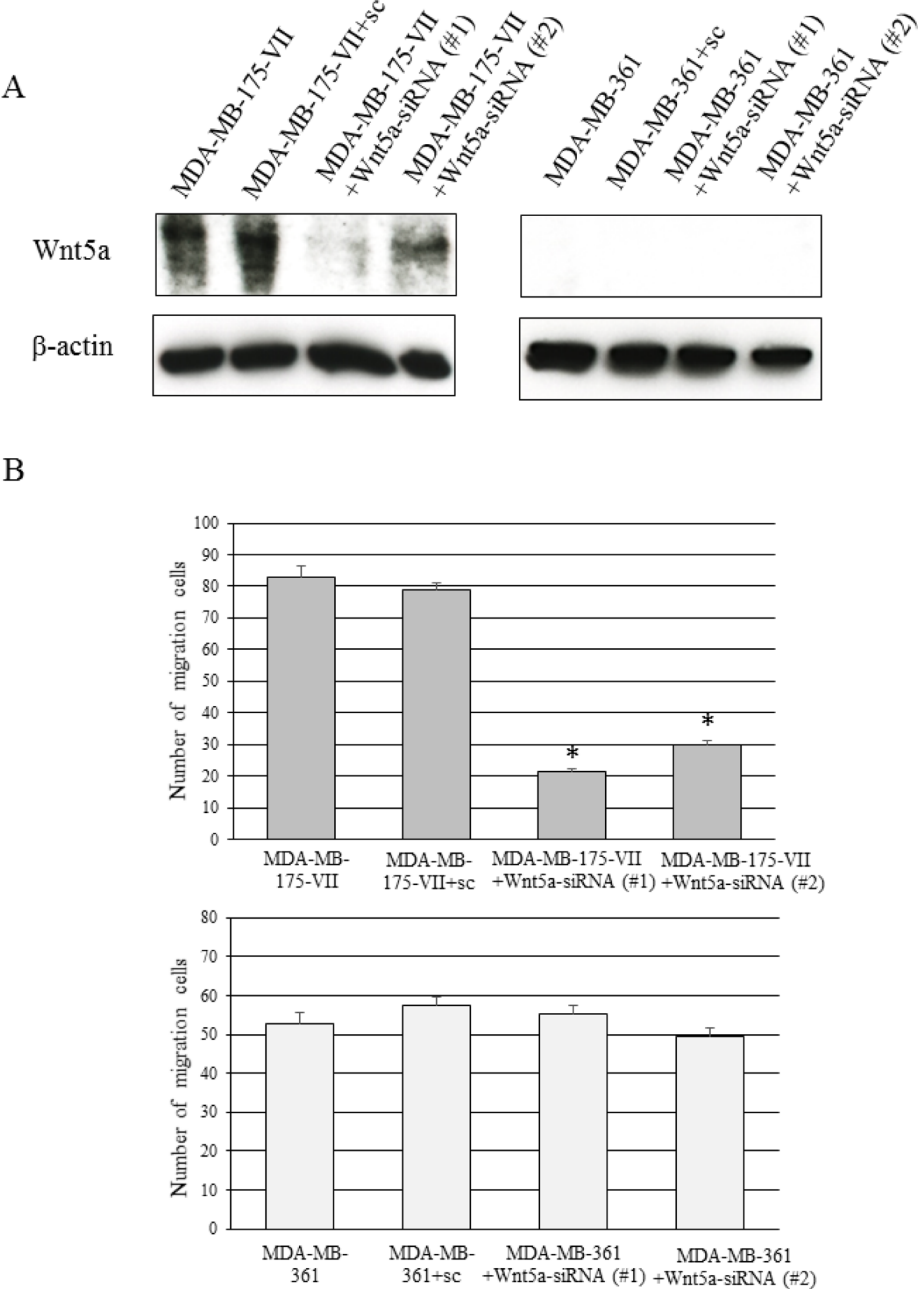
Knockdown of Wnt5a decreases the migratory capacity of MDA-MB-175-VII cells (**A**) The Wnt5a expression of MDA-MB-175-VII and MDA-MB-361 cells transfected with Wnt5a-siRNAs (Wnt5a-siRNA #1 and #2) and sc. (**B**) The migratory capacity of MDA-MB-175-VII and MDA-MB-361 cells transfected with Wnt5a-siRNAs and sc. Data are presented as the mean ± SE of three migration assays. Data were evaluated using the Mann–Whitney *U*-test.

We investigated the endogenous Wnt5a expression in several ER-positive breast cancer cell lines. A strong endogenous Wnt5a expression was observed in MDA-MB-175-VII cells (Figure 4A, Supplementary Figure 1). The knockdown of Wnt5a in MDA-MB-175-VII cells significantly decreased migratory capacity, whereas knockdown of Wnt5a in MDA-MB-361 cells did not decrease migratory capacity (Figure 4B).

### DNA microarray analysis using MCF-7/Wnt5a cells

**Figure 5:**
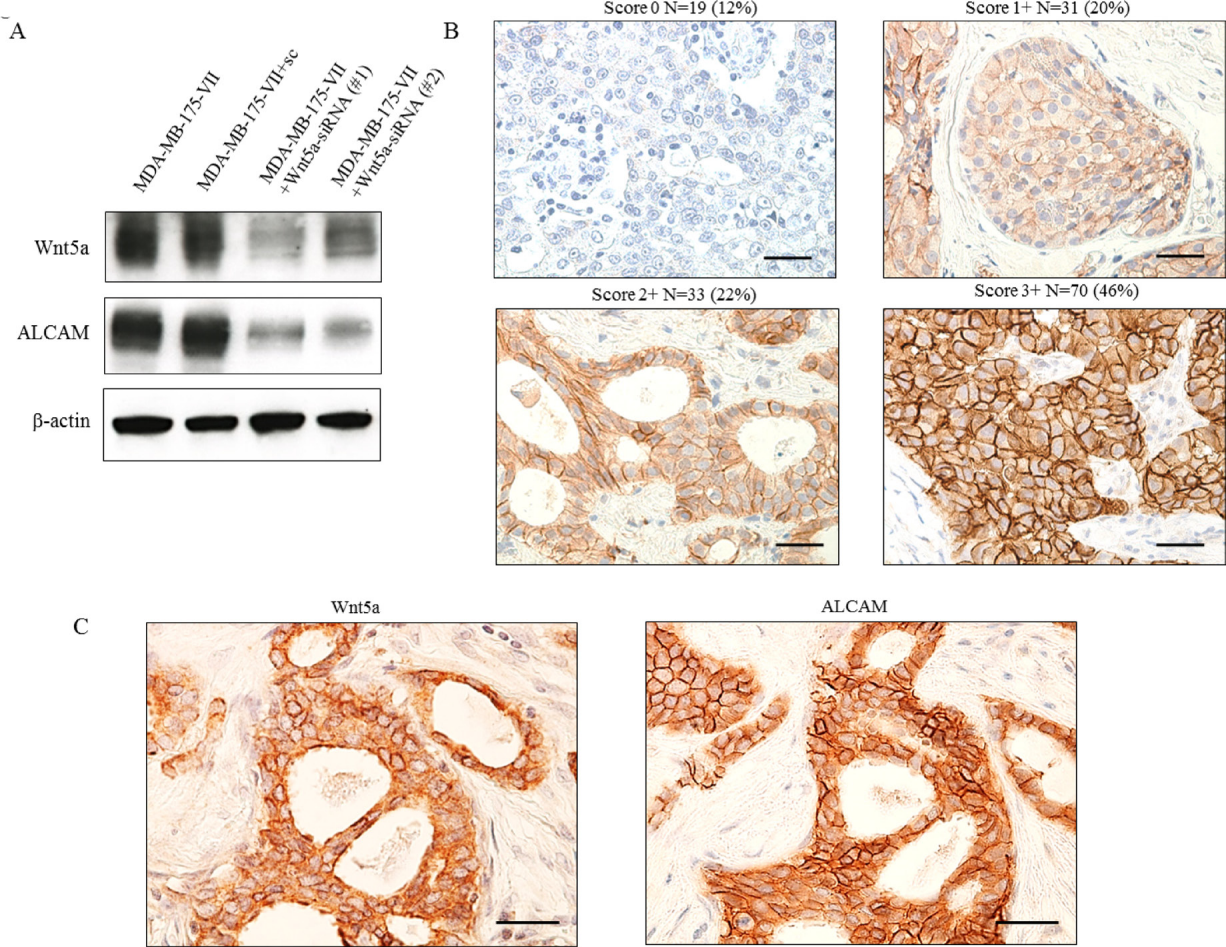
Co-expression of Wnt5a and ALCAM in ER-positive breast cancer tissue (**A**) ALCAM expression was reduced by knockdown of Wnt5a. (**B**) Evaluation of ALCAM expression was scored as 0, 1+, 2+, or 3+. Scoring of ALCAM was performed with the same evaluation as Wnt5a. (**C**) Co-expression of Wnt5a and ALCAM in breast cancer tissue.

To investigate the mechanism by which Wnt5a expression leads to increased migratory activity, we conducted DNA microarray analysis of MCF-7/Wnt5a cells and control cells. We identified several genes upregulated (Table 3) and several downregulated (Supplementary Table 3) by Wnt5a. One of the upregulated genes, ALCAM, is a member of the immunoglobulin superfamily that is localized in the plasma membrane. ALCAM is involved in apoptosis, angiogenesis, migration, and invasion [26–28]. We focused on ALCAM and investigated its protein expression by Western blotting, observing a marked increase of ALCAM in MCF-7/Wnt5a cells and decrease of ALCAM in MCF-7/Wnt5a and MDA-MB-175-VII cells by the knockdown of Wnt5a (Supplementary Figure 2, Figure 5A).

**Table 3:** Genes up-regulated by expression of Wnt5a

			Fold changes (ratio MCF7 Wnt5a/MCF7 control)		
No.	Gene symbol	Gene name	MCF7 Wnt5a ①	MCF7 Wnt5a ②	MCF7 Wnt5a ③
1	ID1	*Inhibitor of DNA binding 1*	3.94	2.08	2.28
2	MAOA	*Amine oxidase A*	2.85	2.26	4.85
3	ALCAM	*Activated leukocyte cell adhesion molecule*	3.25	2.81	3.07
4	AKR1C3	*Aldo-keto reductase family 1 member C3*	3.18	2.18	5.17
5	MARCKS	*Myristoylated alanine-rich C-kinase substrate*	5.93	2.23	10.37
6	CALML5	*Calmodulin-like protein 5*	2.87	2.53	4.10
7	NOL11	*Nucleolar protein 11*	2.85	2.67	2.63
8	STX12	*Syntaxin-12*	3.70	3.84	5.08
9	CCDC104	*Coiled-coil domain-containing protein 104*	2.91	2.79	3.91
10	ITM2B	*Integral membrane protein 2B*	3.13	3.04	3.18
11	CHMP4A	*Charged multivesicular body protein 4a*	3.63	2.96	3.03
12	D2HGDH	*D-2-hydroxyglutarate dehydrogenase*	3.23	3.20	3.76
13	CRK	*Adapter molecule crk*	3.00	3.15	3.00
14	DIABLO	*Direct IAP binding protein with low pI*	3.84	3.15	4.04
15	ZNF446	*Zinc finger protein 446*	2.03	2.05	2.15

### ALCAM expression correlates with Wnt5a expression in ER-positive breast cancer

We examined the expression of Wnt5a and ALCAM in breast cancer specimens. Scoring ALCAM expression as 0–3, we observed that 46% had a score of 3+, which was defined to be positive (Figure 5B). In 153 cases of ER-positive breast cancer, ALCAM was expressed in 69% of Wnt5a-positive breast cancers but only 27% of Wnt5a-negative breast cancers, a statistically significant correlation between Wnt5a and ALCAM expression (Table 4) (κ = 0.444; *P* < 0.001). Serial sections of pathological specimens of invasive breast cancer revealed that Wnt5a was expressed in the cytoplasm, whereas ALCAM was expressed in the plasma membrane (Figure 5C).

**Table 4:** Relationship between Wnt5a expression and ALCAM expression in ER-positive breast cancer

	ALCAM		
Total (*N* = 153)	Negative (*N* = 83)	Positive (*N* = 70)	*P* value
Wnt5a			
Negative (*N* = 85)	62 (73%)	23 (27%)	
Positive (*N* = 68)	21 (31%)	47 (69%)	<0.001

kappa coefficient κ = 0.444. 95% CI.: [0.3013, 0.5869].

### Knockdown of ALCAM also reduces migratory capacity

**Figure 6:**
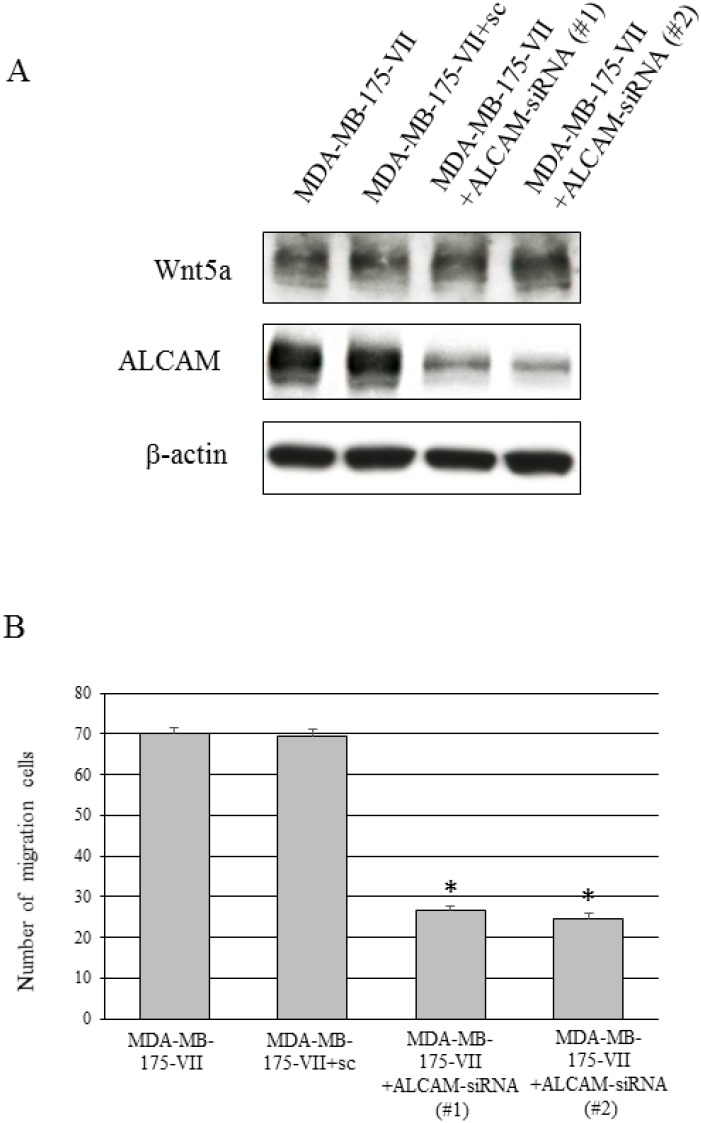
Knockdown of ALCAM decreases the Wnt5a-induced increase in migratory capacity (**A**) Wnt5a and ALCAM expression of MDA-MB-175-VII cells transfected with ALCAM-siRNAs (ALCAM-siRNA #1 and #2) and sc. (**B**) The migratory capacity of MDA-MB-175-VII cells transfected with ALCAM-siRNAs and sc. Data are presented as the mean ± SE of three migration assays. Data were evaluated using the Mann–Whitney *U*-test.

Our data indicate that the knockdown of Wnt5a reduced ALCAM expression and migratory capacity (Figures 3C, 3D and 4B). We next conducted cell migration assays to determine whether inhibition of ALCAM decreases migratory capacity as with the knockdown of Wnt5a. The migratory capacity of MDA-MB-175-VII cells significantly decreased 12 h after the knockdown of ALCAM (Figure 6A, 6B), suggesting that cell migration is regulated via the Wnt5a-ALCAM signaling pathway in ER-positive breast cancer.

## DISCUSSION

We examined nearly 200 specimens using an antibody already confirmed accurate in immunohistochemical analysis of Wnt5a-positive gastric cancers [7]. We observed a high correlation between Wnt5a and ER expression in breast cancers. In addition, our results indicate that Wnt5a-positivity strongly correlated with the presence of lymph node metastasis, lymphatic invasion, vessel invasion, and nuclear grade in ER-positive breast cancer. We also observed that among ER-positive breast cancers, those expressing Wnt5a were more malignant and had a poorer prognosis than those that did not express Wnt5a.

We also investigated the mechanism underlying malignant transformation by Wnt5a. Previous studies have reported that Wnt5a is involved in increased cell proliferation, differentiation, migration, adhesion, and changes in cell polarity [4]. We observed that the migratory capacity of ER-positive breast cancer cells forced to express Wnt5a increased and the knockdown of Wnt5a led to a decrease in migratory capacity, whereas cell proliferation remained unchanged. DNA microarray analysis revealed that ALCAM expression in Wnt5a-positive breast cancer cells is induced by Wnt5a. ALCAM expression in breast cancer is reported to correlate with ER expression, lymph node metastasis, distant metastasis, and a poor prognosis [26, 30, 31]. Activation of the β-catenin-independent pathway induces ALCAM expression, and JNK located downstream of Wnt5a induces ALCAM expression [32]. We explored the function of Wnt5a and ALCAM on cell migration in ER-positive breast cancer and found that the knockdown of Wnt5a reduced ALCAM expression and cell migration. Furthermore, the knockdown of ALCAM also showed the reduction of cell migration, suggesting that cell migration is regulated via the Wnt5a-ALCAM signaling pathway in ER-positive breast cancer. Our investigation of breast cancer tissues also revealed that Wnt5a expression correlates with ALCAM expression in ER-positive breast cancers, indicating that Wnt5a-positive/ALCAM-positive breast cancers form a biologically distinct subgroup of ER-positive breast cancers.

Wnt5a expression has been shown to correlate with tumor progression in a variety of cancers. However, the function of Wnt5a has been reported as both a tumor suppressor and a tumor promotor in breast cancer. The loss of Wnt5a was found to be an indicator of poor prognosis in a study of whole breast cancers, including different subtypes [17]. However, accurate assessment of Wnt5a function in different subtypes is difficult because mutations in the signaling molecules differ between different subtypes. Therefore, it is important to determine which subtypes express Wnt5a. The majority of the previous reports on Wnt5a-positive breast cancer classified these cancers based solely on the presence or absence of Wnt5a expression, without regard to subtype. In addition, the number of cases studied in these reports was small, and there was not a suitable antibody available for immunohistochemical analysis of tissues.

Wnt5a overexpression was observed in ER-positive breast cancers with a mutation in *PIK3CA* [19]; *PIK3CA* mutations are seen in approximately 30% of ER-positive breast cancers and cause resistance to therapy [33, 34]. Together with these reports, our results suggest that Wnt5a is involved in the malignant transformation and/or relapse of ER-positive breast cancer. Wnt5a-Ror2 signals increase the expression of receptor activator of nuclear factor-κB (RANK) in osteoclast precursors by activating JNK and recruiting c-Jun, thereby enhancing RANK ligand (RANKL)-induced osteoclastogenesis [35]. Interestingly, all patients with recurrent breast cancer in this study had bone metastasis, suggesting that Wnt5a plays a role in bone metastasis in breast cancer.

Increased Wnt5a expression has been observed in ER-positive breast cancers with *PIK3CA* mutations [19]. Thus, we propose that mutations in *PIK3CA* induce Wnt5a expression, which activates JNK to induce ALCAM expression, causing malignant transformation of ER-positive breast cancer cells. It is unknown whether Wnt5a expression is induced by activation of PI3K/AKT/mTOR signaling caused by *PIK3CA* mutation in ER-positive breast cancers. This question will be the focus of our future studies.

In conclusion, Wnt5a induces malignant transformation in ER-positive breast cancers. Increased invasiveness resulting from upregulated ALCAM expression suggested the mechanism whereby Wnt5a induces malignant transformation. Wnt5a may be useful as a predictor of malignancy, a therapeutic target, and a prognostic indicator in ER-positive breast cancer.

## MATERIALS AND METHODS

### Sample selection

Patients with invasive breast cancer treated surgically between January 2011 and February 2014 at Hiroshima University Hospital in Japan were sequentially enrolled in this study. Patients treated with neoadjuvant chemotherapy were excluded. This study was approved by the institutional review board of Hiroshima University Hospital (No. 926), and all participants provided written informed consent for the use of their tissue specimens. Each participant's medical information was obtained from medical records.

### Immunohistochemistry

Immunohistochemistry was performed as previously described [22]. Antigen retrieval was made using proteinase treatment (Proteinase K S3004; Dako, Carpinteria, California, USA) for 2 min and 30 s in room air. The sections were reacted with the polyclonal anti-Wnt5a antibody, a kind gift from Prof. Akira Kikuchi (Department of Molecular Biology and Medicine, Osaka University, Suita, Japan) at 4° C overnight. Activated leukocyte cell adhesion molecule (ALCAM) was stained with anti-ALCAM antibody (clone MOG/07; Leica Biosystems, Wetzlar, Hesse, Germany). Antigen retrieval was made using heat-treatment for 20 min at 98° C in citrate buffer (pH 6.0). Wnt5a and ALCAM expression were scored as 0, 1+, 2+, or 3+, taking into consideration staining proportion and intensity. The Wnt5a and ALCAM scores were assessed as follows: 0, no staining or staining in ≤10% of invasive tumor cells; 1+, weak staining in >10% of invasive tumor cells; 2+, moderate staining in >10% of invasive tumor cells or strong staining in >10% and ≤30% of invasive tumor cells; 3+, strong staining in >30% of invasive tumor cells. A score of 3+ was defined as positive for both Wnt5a and ALCAM. The scoring was independently conducted by two investigators who had no knowledge of the patients’ clinical data. An automatic staining machine was used for immunohistochemical staining of the following biomarkers: ER, PgR (SP-1, monoclonal antibody; Ventana Medical Systems, Tucson, Arizona, USA), HER2 (polyclonal antibody; Dako), and Ki-67 (30-9, monoclonal antibody; Ventana Medical Systems). A proportion of nuclear staining of ER and PgR ≥1% was considered positive expression. HER2 expression was evaluated accordingly as previously described [29].

### Cell culture and transfection

MCF-7 cells were obtained from American Type Culture Collection (Manassas, Virginia, USA) and were grown in RPMI-1640 medium supplemented with 10% fetal bovine serum. MCF-7 cells were transiently transfected with pPGK-neo/Wnt5a using Lipofectamine LTX with PLUS reagent (Life Technologies, Carlsbad, California, USA). pPGK-neo/Wnt5a was a kind gift from Prof. Akira Kikuchi. MCF-7 cells stably expressing mouse Wnt5a (MCF-7/Wnt5a) were generated by selection with 200 μg/mL G418.

We sough breast cancer cells expressing ER, Wnt5a, and ALCAM endogenously to address the mechanism by which ALCAM mediates the ability of Wnt5a to promote migration of ER-positive breast cancer cells. MDA-MB-175-VII, MDA-MB-361, ZR-75-30, ZR-75-1, and CAMA-1 cells were obtained from American Type Culture Collection. MDA-MB-175-VII, MDA-MB-361, and CAMA-1 cells were grown in Leibovitz's L-15 medium supplemented with 10% fetal bovine serum, Leibovitz's L-15 medium supplemented with 20% fetal bovine serum, and Eagle's minimum essential medium supplemented with 10% fetal bovine serum, respectively. ZR-75-30 and ZR-75-1 cells were grown in RPMI-1640 medium supplemented with 10% fetal bovine serum.

### siRNA

In analyses with small interfering RNAs (siRNAs) for Wnt5a, the human Wnt-5a mRNA target sequences, 5ʹ-TATCAATTCCGACATCGAATT-3ʹ and 5ʹ-UAUCAAUUCCGACAUCGAATT-3ʹ were used (Life Technologies). For ALCAM, the human ALCAM mRNA target sequences, 5ʹ-GCAUAUGGAGAUACCAUUATT-3ʹ and 5ʹ-GGACCUCGGUAAUAUGGAATT-3ʹ were used (Life Technologies). To transfect siRNA into MCF-7 cells expressing Wnt5a, trypsinized MCF-7 cells were suspended in Opti-MEM (Life Technologies) at 10^6^ cells per 100 μL, siRNA was added (90 pmol), and cells were mixed with RNAi max (Life Technologies) in Opti-MEM.

### Cell migration assay

To assess cell migration activity, a two-chamber transmigration assay was performed as previously described [36, 37]. MCF-7/Wnt5a cells (5.0 × 10^4^ cells), 5.0 × 10^4^ MCF-7/Wnt5a cells + scramble (sc), 5.0 × 10^4^ MCF-7/Wnt5a + Wnt5a-siRNA, 5.0 × 10^4^ MCF-7/Wnt5a + ALCAM-siRNA, and 5.0 × 10^4^ MCF-7 cells with 500 μL RPMI-1640 medium were seeded into the upper chamber, and 750 μL NIH3T3-conditioned medium and 750 μL RPMI-1640 medium (10% FBS) was added to the lower chamber of a 12-well Multi Well Plate (BD Biosciences, San Jose, California, USA). Cells were incubated for 12 h at 37° C. The migrated cells on the underside of the upper chamber were stained with phalloidin (P1951; Sigma-Aldrich, St. Louis, Missouri, USA) and counted per 10 high-power fields (×200) using a fluorescence microscope (FV1000; Olympus, Tokyo, Japan). These experiments were performed at least three times in each cell line.

### Cell proliferation assay

The control MCF-7 and MCF-7/Wnt5a cells were seeded at densities of 1 × 10^5^/mL as previously described [8]. Cells were enumerated on the incubated days.

### Gene microarray analysis

For the Oligo DNA microarray analysis, cells were washed with phosphate-buffered saline once and collected with Buffer RLT (Qiagen, Hilden, North Rhine-Westphalia, Germany). Total RNA was extracted from cells using the RNeasy mini kit (Qiagen). The 3D-Gene Human Oligo chip 25 k (Toray Industries, Tokyo, Japan) was used. For efficient hybridization, this microarray has 3-dimensions constructed with a well as the space between the probes and cylinder-stems, with 70-mer oligonucleotide probes on the top. Total RNA was labeled with Cy5 using the Amino Allyl MessageAMP II aRNA Amplification Kit (Applied Biosystems, Foster City, California, USA). The Cy5-labeled aRNA pools were placed in hybridization buffer and allowed to hybridize for 16 h using the supplier's protocols (www.3d-gene.com). The hybridization signals were obtained using the 3D-Gene Scanner (Toray Industries) and processed by 3D-Gene Extraction (Toray Industries). Detected signals for each gene were normalized using the global normalization method, with the median of the detected signal intensity adjusted to 25.

### Materials and chemicals

Anti-Wnt5a/b antibody was purchased from Cell Signaling Technology (Beverly, Massachusetts, USA). Anti-β-catenin and anti-ALCAM antibodies were purchased from BD Biosciences and Leica Biosystems, respectively. Anti-ER and anti-ErbB 2 antibodies were purchased from Abcam. Anti-β-actin and anti-cyclin D1 antibodies were purchased from Sigma-Aldrich.

### Statistical analysis

The correlation between immunohistochemical determination of Wnt5a expression and clinicopathologic factors in tumor tissues was analyzed using the Chi-square test. The correlation between Wnt5a and ALCAM expression was analyzed by kappa coefficient. The Mann–Whitney *U*-test was used to compare cell migration. *P* values < 0.05 were considered statistically significant. All calculations were performed using SPSS version 20.

## SUPPLEMENTARY MATERIALS FIGURES AND TABLES



## Abbreviations

JNK: c-Jun N-terminal kinase
*PIK3CA*: phosphatidylinositol-4,5-bisphosphate 3-kinase catalytic subunit alpha
FAK: focal adhesion kinase
Rac: RAS-related C3 botulinus toxin substrate
Ror: receptor tyrosine kinase-like orphan receptor
MCF-7: Michigan Cancer Foundation7
RPMI: Roswell Park Memorial Institute medium
pPGK-neo: plasmid phosphoglycerate kinase 1 promoter-neomycin
PI3K: phosphatidylinositol 3-kinase
mTOR: mammalian target of rapamycin
SE: standard error.
