# Supplementary material for: Wnt5a-induced cell migration is associated with the aggressiveness of estrogen receptor-positive breast cancer
Source: Oncotarget. 2018 Apr 20;9(30):20979–92. doi: 10.18632/oncotarget.24761 (PMC5940389; doi:10.18632/oncotarget.24761)
Supplement: Supplementary file 1 [file oncotarget-09-20979-s001.pdf]

Wnt5a-induced cell migration is associated with the aggressiveness of estrogen receptor-positive breast cancer

SUPPLEMENTARY MATERIALS

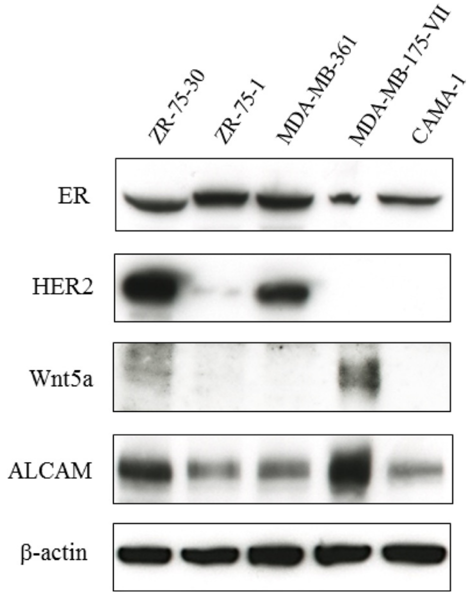

Supplementary Figure 1:Wnt5a expression in ER-positive breast cancer cell lines. Endogenous expression of Wnt5a an ALCAM in ER-positive breast cancer cell lines.

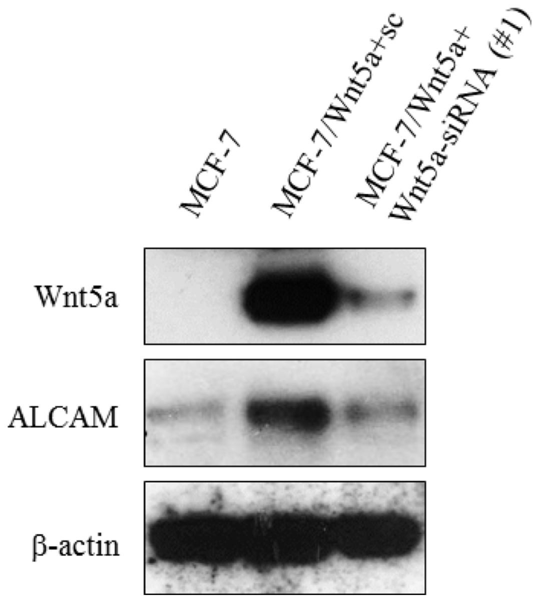

Supplementary Figure 2: Wnt5a regulates ALCAM expression. ALCAM expression was induced by expression of Wnt5a and reduced by knockdown of Wnt5a in MCF-7 cells..

Supplementary Table 1: Relation between Wnt5a expression and clinicopathological factors in breast cancer

	Wnt5a expression			
	**Total (*N* = 178)**	**Negative (*N* = 109)**	**Positive (*N* = 69)**	***P***
value				
Age (median, range)		62, 35–86	58, 34–87	0.148
Age, *N* (%)				
≤45	31 (17)	16 (15)	15 (22)	
>45	147 (83)	93 (85)	54 (78)	0.226
Menopausal status, *N* (%)				
Premenopausal	63 (35)	32 (29)	31 (45)	
Postmenopausal	115 (65)	77 (71)	38 (55)	0.034
Histology, *N* (%)				
Invasive ductal carcinoma	158 (89)	97 (89)	61 (89)	
Invasive lobular carcinoma	6 (3)	3 (3)	3 (4)	
Others	14 (8)	9 (8)	5 (7)	0.841
Tumor size, *N* (%)				
pT1 ≤ 20 mm	124 (70)	83 (76)	41 (59)	
pT2/pT3 > 20 mm	54 (30)	26 (24)	28 (41)	0.018
Lymph node metastasis, *N* (%)				
Negative	119 (67)	88 (81)	31 (45)	
Positive	59 (33)	21 (19)	38 (55)	0.018
ER, *N* (%)				
Negative	25 (14)	24 (22)	1 (1)	
Positive	153 (86)	85 (78)	68 (99)	<0.001
PgR, *N* (%)				
Negative	38 (21)	34 (31)	4 (6)	
Positive	140 (79)	75 (69)	65 (94)	<0.001
HER2, *N* (%)				
Negative	159 (89)	96 (88)	63 (91)	
Positive	19 (11)	13 (12)	6 (9)	0.496

Supplementary Table 2: Clinicopathological factors of recurrent ER-positive breast cancer

Case	Age at operation	Tumor size (mm)	Nuclear grade	Lymph node metastasis (n)	Lymphatic invasion	Vessel invasion	ER (%)	PgR (%)	HER2	Ki-67 (%)	Subtype	Wnt5a	ALCAM	Sites of recurrence
1	34	35	3	0	Positive	Negative	95	100	0	43	Luminal	Positive	Positive	Liver, pleura, bone, Lymphangiosis carcinomatosa
2	62	40	3	1	Negative	Negative	100	0	1+	17	Luminal	Positive	Positive	bone
3	79	45	3	6	Positive	Negative	100	75	1+	63	Luminal	Positive	Positive	bone, pleura
4	41	70	3	1	Positive	Negative	90	90	3+	76	Luminal-HER2	Positive	Negative	bone

Supplementary Table 3: Genes down-regulated by expression of Wnt5a

			Fold changes (ratio MCF7 Wnt5a/MCF7 control)		
**No.**	**Gene symbol**	**Gene name**	**MCF7 Wnt5a ①**	**MCF7 Wnt5a ②**	**MCF7 Wnt5a ③**
1	OASL	*2'-5'-oligoadenylate synthetase-like protein*	0.08	0.23	0.05
2	CXCR4	*C-X-C chemokine receptor type 4*	0.32	0.48	0.25
3	UBE2L6	*Ubiquitin/ISG15-conjugating enzyme E2 L6*	0.24	0.32	0.16
4	ISG15	*Interferon-induced 15 kDa protein*	0.11	0.19	0.14
5	SLC39A8	*Solute carrier family 39 member 8*	0.29	0.33	0.38
6	EIF2AK2	*Eukaryotic translation initiation factor 2-alpha kinase 2*	0.27	0.38	0.31
7	PCP4	*Purkinje cell protein 4*	0.42	0.24	0.08
8	NCAM2	*Neural cell adhesion molecule 2*	0.34	0.35	0.20
9	IFI27	*Interferon alpha-inducible protein 27*	0.01	0.08	0.04
10	SP110	*Sp110 nuclear body protein*	0.33	0.28	0.48
11	OAS3	*2'-5'-oligoadenylate synthetase 3*	0.11	0.12	0.12
12	STAT1	*Signal transducer and activator of transcription 1*	0.22	0.32	0.34
13	RAB31	*Ras-related protein Rab-31*	0.31	0.49	0.28
14	ANXA3	*Annexin A3*	0.42	0.44	0.32
15	IFITM1	*Interferon-induced transmembrane protein 1*	0.04	0.17	0.09
